# The Structure and Development of Bone

**Published:** 1853-04

**Authors:** John Tomes, Campbell De Morgan

**Affiliations:** Surgeon Dentist to the Middlesex Hospital; Surgeon to the Middlesex Hospital.


### ARTICLE XIII.

*The Structure and Development of Bone.* By JOHN TOMES, F. R. S., Surgeon Dentist to the Middlesex Hospital, and CAMPBELL DE MORGAN, Surgeon to the Middlesex Hospital.

IN this communication, the authors, after having briefly noticed the intimate structure of perfect bone as commonly recognised, proceed to the description of certain points connected with its structure and development, which they believe to have been hitherto entirely overlooked or only partially recognised.

These points have been arranged under the following heads:

1. The Haversian and other canals of bone.
2. The laminæ of bone.
3. The lacunæ.
4. Haversian systems.
5. Ossified cartilage of joints.
6. Ossified cells.
7. Bone tissue.
8. Development of bone in temporary cartilage.
9. Growth of bone.

1. *Haversian and other Canals of Bone.*—Besides the Haversian canals, the authors have pointed out that there are found in bone sections spaces of an entirely different character, irregular in shape, and with an irregular festooned margin. Their margins correspond in outline with those of one or more Haversian systems, and precede in many instances the formation of those systems. These spaces, produced by absorption, are called by the authors *Haversian spaces.* Unlike the Haversian canals which are surrounded by their own laminæ, these spaces are bounded by parts of several systems which have been encroached on by the process of absorption.

In examining various sections, or different parts of the same section, many of these spaces will be found which have become partially or entirely occupied by Haversian systems. They are found in the bone of subjects of all ages. The fact of removal of old tissue and replacement by new, which has been hitherto only assumed, is thus demonstrated.

2. *Laminæ of Bone.*—Lamination is shown to be a constant character of mammalian bone; each lamina, when highly developed, is found to consist of a dark granular, and of a transparent part. The external margin of the outermost lamina of each Haversian system is irregularly indented and corresponds with the outline of a pre-existing Haversian space; while its internal margin and all the succeeding laminæ are regular in outline.

The laminæ are found as a general rule to surround their canal, which is usually placed in the centre of them. But sometimes the canals are eccentric, in which case either the laminæ on one side, though still surrounding the canal, are broader, or more are developed on one side than on the other. The laminæ next to the perfected Haversian canal however is always complete, and is often composed of a transparent structureless tissue, like that which encircles the Haversian canals of the stag's antler at the time of shedding.

The presence of interstitial laminæ is readily accounted for; they are in fact the remains of pre-existing Haversian systems, or circumferential laminæ, parts of which have been removed by absorption.

The circumferential laminæ are not so constantly present as is generally described, and seldom entirely surround the shaft of a long bone. When present, they seem to indicate that the bone is nearly stationary in its growth. They are frequently intersected by numerous Haversian spaces and systems, so as at length to assume the characters of interstitial laminæ.

3. *Lacunæ.*—In young bone the lacunæ are more abundant, larger, and have more numerous canaliculi; in older bone they may exist without canaliculi, or the canaliculi and great part of the lacunæ themselves may be filled up with solid matter, so as to leave only a small space in the centre of the latter. The lacunæ and canaliculi are shown to have distinct walls.

In the circumferential laminæ are frequently found elongated tubes which the authors regard as modifications of lacunæ; they run obliquely across the laminæ, generally in bundles. They frequently form communications with the canaliculi. In transverse section they are seen to have proper walls.

4. *Haversian Systems.*—The authors have here pointed out that the anastomosis of the canaliculi of adjoining systems is rare in newly developed systems, but is very common in those of greater age. It has been seen too that it not unfrequently happens that a series of Haversian systems is contained within a common series of surrounding laminæ. Sometimes the Haversian systems are rendered quite solid by the narrowing of the Haversian canal and ultimate development of a mere lacuna in the centre of the system. The more recently developed Haversian systems, which occupy the Haversian spaces before described, are seen to be darker in color than the older ones, from the greater abundance of canaliculi, and the more general granularity of the tissue.

5. *Ossified Articular Cartilage.*—This structure the authors have found in all the joints which they have examined, in the lower jaw, amongst others, where Kölliker failed to detect it. Towards the bone the tissue becomes in general granular and of a brownish color, and usually there is a distinct line of demarcation between the bone and the ossified cartilage; but sometimes they graduate insensibly the one into the other.

Towards the articular surface the margin is even and regular; but towards the bone it is deeply indented, from the bone advancing into it by rounded projections. Hence the articular cartilage varies in thickness. The authors believe that this, so far from being an indication of imperfect development, is in reality an evidence of design, and intended to give an uniform and unyielding surface for the cartilage to rest upon.

6. *Ossified Cells.*—In the bones of aged people it is frequently observed that they become light and spongy, and after maceration contain a white powder in the cancellated structure. This powder the authors have found to be composed mainly of ossfied nucleated cells, either detached or held together in masses. They are spherical, and contain a dark granular nucleus, which is surrounded by a thick transparent wall.

If portions of the cancelli be examined, they will be found to have similar cells adherent to their surfaces, or to those of the Haversian canals, with here and there canaliculi of adjoining lacunæ shooting into them, while the nuclei have themselves assumed the form of lacunæ. Similar cells may be found imbedded in parts of most sections of bone. In order to see this condition clearly, it is desirable that the sections and the loose cells should be mounted in Canada balsam.

7. *Bone Tissue.*—The views generally entertained with regard to the ultimate structure of bone tissue are, the older one, that it consists of an aggregation of granules in a transparent matrix; and that which has been more recently put forward by Dr. Sharpey, that in many cases it is composed of ossified decussating fibres.

The authors have satisfied themselves that the ultimate structure of bone tissue is composed of minute granules or granular bodies imbedded in a clear or subgranular matrix; and that the appearance of fibres is due in many cases to the mode of illumination. By transmitted light passing through them in the long axis of the microscope the preparations show a granular or a structureless appearance, or alternations of a granular, and structureless part. But under an oblique light passing from one side only an appearance of minute flat fibres presents itself. This takes place even in the isolated cells of old bone, or in developing young bone. This appearance is most marked over the lacunæ and canaliculi. But if a part which thus appears fibrous be viewed under a light passing obliquely from all sides, as is effected by a Gillett's achromatic condenser, the fibres disappear, and we see only a granular appearance, with some tendency to arrangement in the granules. The fibrous appearance is in fact due to the shadows cast from the less transparent parts when the light passes obliquely, just as in the navicula the dots are replaced by lines. In thin sections torn from bone which has been macerated in acid, a reticulated appearance, similar to that figured by Dr. Sharpey, may be seen, only however when the object is slightly out of focus, or the light oblique and from one side. By careful adjustment of the object-glass and of the illuminating apparatus, this appearance may be shown to depend on the presence of the canaliculi.

8 and 9. *Development and Growth of Bone.*—The early condition of cartilage, and the changes which take place in it and in the cartilage cell before ossification, are particularly described; and also the mode by which they multiply and arrange themselves by segmentation, so that a long column or cluster of cells represents an original cell, the walls of which have coalesced with the surrounding hyaline tissue. The cells at the same time enlarge individually as they approach the point where ossification is going on, encroaching on the hyaline substance so as in many cases only to leave a fine line of intercolumnar tissue, or even to cause it to disappear altogether. The nucleus at the same time enlarges considerably, while the cell wall becomes thickened internally, until in the end it reaches the nucleus, which then becomes imbedded in firm tissue. Other changes now take place: either several cells are thrown into one cavity by the absorption of their contiguous walls, leaving the nuclei free in the common cavity; or the nucleus continues to occupy its parent cell, and sends off small processes, which extend outwards to the cell wall. At this stage the nucleus may be sometimes detached with the processes entire, but generally it is adherent, and may be seen to have become a lacuna with a central cavity and canaliculi; in addition to which a nucleus may be seen to occupy its interior; it has in fact become a nucleated cell, designated by the authors "*granular cell.*" The entire cell may now be detached from the intercolumnar tissue in which it lies.

The granular condition of the intercolumnar tissue and of the cell itself often renders the observation of this stage very difficult; but in rickety bone it is very readily shown, as in this disease there is a tendency for the cells to assume their permanent form before the deposit of bone-earth in any considerable quantity. To cells thus composed of an outer thickened cell wall and an inner granular cell (the cartilage nucleus of authors) which contains within it a nucleus (the nucleolus of writers,) which stands in the relation of a nucleus to the future lacuna, the authors have given the name of "*lacunal cells*," while the term granular cell has been applied to that which is usually designated the nucleus. In transverse sections of bone immediately below the line of ossification, the lacunal cells may be seen presenting different characters under different circumstances. Where two cells come into contact, the processes or canaliculi may be seen extending across from one to the other; but where the cell is surrounded by intercolumnar tissue, the processes are short and do not extend beyond the walls of their own cell; or if cells join at one point while the remainder is invested with intercolumnar tissue, the canaliculi will anastomose at the point of junction; while elsewhere they are few, short, and do not extend beyond the cell.

In the further process of development the cells and intercolumnar tissue become fused together so as no longer to be recognised as distinct parts; and the granular cell appears as a perfect lacuna with a large cavity and numerous large canaliculi. To bone in this condition the term primary bone has been applied. It speedily however undergoes a change preparatory to the formation of the more permanent secondary bone. Here and there in the line of ossification portions are removed by absorption, the spaces left being filled with small somewhat granular cells, lying in a transparent blastema, and through the agency of which the absorption has been in all probability effected. It would appear as though the cells grew at the expense of the surrounding tissue. These spaces correspond entirely to the Haversian spaces before described; and in them the secondary bone is in the first instance formed. The process of formation of secondary bone appears to be everywhere essentially the same, whether in the absorbed spaces, or on the surfaces, or in the membranes of the fœtal cranium, except that in the two latter cases there is a pre-existing fibrous tissue, which, before ossification begins, undergoes a change similar to that which occurs in the bone itself and is converted into a cellular mass. So that at the border where ossification is advancing there is only an arrangement of cells; while a little beyond that point the cells have fibrous tissue abundantly mixed up with them; and there is in fact a resemblance to fibrous tissue in an early state of formation. The formation of perfect bone is effected by means of cells, perhaps identical with those which are found replacing the previous tissue, but at all events undistinguishable from them by any microscopical characters. To these cells, which take part in the formation of bone, the authors have given the name of "*osteal cells.*"* In the case of laminated bone they arrange themselves side by side, and, together with the transparent blastema in which they lie, become impregnated with ossific matter, and permanently fused with the bone tissue with which they lie in contact. By the linear arrangement of these osteal cells lamination is produced. In the case of non-laminated bone the cells are simply ossified without arrangement. Lying amongst the osteal cells will be seen some which have accumulated around them a quantity of tissue which forms a thick investment to them; they then become granular, and take on, in every respect, the characters of a lacunal cell. These are found deposited at intervals along the line of ossification and becoming blended with the general mass; the granular cell remaining as a lacuna, and sending out processes amongst the cells in all directions. In old bone the cell character is in great part lost by a general blending of the constituents, but may in many specimens be still here and there recognised. Many instances are given in support of the conclusion that absorption of bone and of dental tissue is effected directly through the influence of cells, but these are necessarily excluded from this abstract; indeed it is impossible to give any other than a very imperfect account of the contents of the paper within the prescribed limits, especially as the numerous illustrations which accompany the paper cannot be made use of.

* The views here brought forward of the removal and replacement of tissue through the agency of cells are, so far as the authors know, entirely new; and may have an important bearing on many points of physiology and pathology. Indeed, this is perhaps the first time that the fact (which has been generally assumed) of the entire absorption of tissue in the processes of nutrition, and its replacement by new tissue, has been demonstrated.

